# Impact of a Partial Smoke-Free Legislation on Myocardial Infarction Incidence, Mortality and Case-Fatality in a Population-Based Registry: The REGICOR Study

**DOI:** 10.1371/journal.pone.0053722

**Published:** 2013-01-23

**Authors:** Fernando Agüero, Irene R. Dégano, Isaac Subirana, Maria Grau, Alberto Zamora, Joan Sala, Rafel Ramos, Ricard Treserras, Jaume Marrugat, Roberto Elosua

**Affiliations:** 1 Department of Research in Inflammatory and Cardiovascular Disorders (RICAD), IMIM (Hospital del Mar Medical Research Institute), Barcelona, Spain; 2 Department of Preventive Medicine and Public Health, Parc de Salut Mar-Pompeu Fabra University-Public Health Agency of Barcelona, Barcelona, Spain; 3 Epidemiology and Public Health CIBER (Centros de Investigación Biomédica en Red), Barcelona, Spain; 4 Department of Internal Medicine, Hospital Comarcal de Blanes, Girona, Spain; 5 Department of Cardiology, Hospital Josep Trueta, Girona, Spain; 6 Department of Cardiovascular Research, IDIAP JGol (Institut d'Investigació en Atenció Primària Jordi Gol), Girona, Spain; 7 Departament de Salut, Generalitat de Catalunya, Barcelona, Spain; Virginia Commonwealth University, United States of America

## Abstract

**Background and Objective:**

Coronary heart disease (CHD) is the leading cause of death, and smoking its strongest modifiable risk factor. Our aim was to determine the impact of the Spanish 2006 partial smoke-free legislation on acute myocardial infarction (AMI) incidence, hospitalization and mortality rates, and 28-day case-fatality in Girona, Spain.

**Methods:**

Using a population-based registry (the REGICOR Study), we compared population incidence, hospitalization, and mortality rates, and 28-day case-fatality in the pre- and post-ban periods (2002–2005 and 2006–2008, respectively) by binomial regression analysis adjusted for confounding factors. We also analyzed the ban's impact on the outcomes of interest using the AMI definitions of the American Heart Association (AHA)/European Society of Cardiology (ESC) and the World Health Organization (WHO)-Monitoring trends and determinants in cardiovascular diseases (MONICA).

**Results:**

In the post-ban period, AMI incidence and mortality rates significantly decreased (relative risk [RR] = 0.89; 95% confidence interval [CI] = 0.81–0.97 and RR = 0.82; 95% CI = 0.71–0.94, respectively). Incidence and mortality rates decreased in both sexes, especially in women, and in people aged 65–74 years. Former and non-smokers (passive smokers) showed diminished incidence rates. Implementation of the ban was not associated with AMI case-fatality. Models tended to be more significant with the WHO-MONICA than with the AHA/ESC definition.

**Conclusions:**

The 2006 Spanish partial smoke-free legislation was associated with a decrease in population AMI incidence and mortality, particularly in women, in people aged 65–74 years, and in passive smokers. These results clarify the association between AMI mortality and the enactment of a partial smoke-free legislation and reinforce the effectiveness of smoking regulations in preventing CHD.

## Introduction

Smoking and second-hand smoke (SHS) are major and preventable public health hazards [1] and risk factors for coronary heart disease (CHD) [2], [3], the single most common cause of death and morbidity worldwide. Public health authorities have implemented various policies such as smoke-free legislations to reduce the burden of CHD. Analysis of the effects of smoke-free legislations in different populations has shown substantial reduction in hospital admissions for acute coronary syndrome after the enactment of the ban [4]–[15].

However, most previous studies used data from hospital-based registries and only two studies have analyzed the impact of smoke-free legislations in population-based registries [6], [7]. This is particularly important because about two thirds of fatal acute myocardial infarction (AMI) events occur before reaching the hospital [16]. Therefore, a population-based registry should provide a broader depiction of the disease and of the potential impact of smoke-free legislations. In addition, only a few studies have analyzed the association between smoke-free legislation enactment and AMI mortality [17]–[20], with contradictory results. No study has examined the association between the enactment of a smoke-free legislation and AMI 28-day case-fatality.

In January 2006, Spain implemented smoking regulations (Law 28/2005) that included a ban on advertising, a reduction in sales outlets, and a partial smoke-free legislation which banned smoking in all indoor public places and workplaces but allowed some exceptions in hospitality venues. Hospitality venues >100 m2 could be smoke-free or have a smoking section up to 30% of the total area while venues <100 m2 could decide to be smoke-free or to allow smoking without restrictions [21]. Studies of the immediate impact of the 2006 partial smoke-free legislation reported a decrease in AMI hospitalization rates in Barcelona from 2004 to 2006 [13], and a decrease in adjusted AMI mortality rates in Spain from 2004 to 2007 [20]. In January 2011, Spain passed a new law (Law 42/2010) that amended the previous of 2005 and banned smoking in all workplaces with no exceptions in the hospitality sector.

The main aim of this study was to analyze the impact of the 2006 Spanish partial smoke-free legislation on AMI incidence, hospitalization and mortality rates, and 28-day case-fatality in a population-based registry and a hospital-based registry. The secondary aims were to analyze the effect of the partial smoke-free legislation on different subgroups of the population and to compare results using the AMI definitions of the American Heart Association (AHA)/European Society of Cardiology (ESC) definition, in use since 2000, and the World Health Organization (WHO)-Monitoring trends and determinants in cardiovascular diseases (MONICA) in this particular setting.

## Methods

### Ethics statement

The REGICOR study was approved by the IMAS (Institut Municipal d'Assistencia Sanitaria) Ethics Committee and has been performed in accordance with the ethical standards laid down in the 1964 Declaration of Helsinki and its later amendments. No informed consent was obtained from the participants. The REGICOR study is a population-based myocardial infarction registry and one of the requirements of a population registry is to be exhaustive to guaranty the validity of the incidence estimates obtained, thus all cases have to be included. To respect and guarantee the confidentiality of the patients the investigators did not have access to individual confidential data and the data were analyzed anonymously.

### Study design

This was a before-and-after observational study using a population-based AMI registry, the REGICOR Study, to analyze the effect of 2006 Spanish partial smoke-free legislation on AMI incidence and mortality outcomes. The REGICOR (*REgistre GIroní del COR*; Girona Heart Registry) Study, conducted in six counties of the Girona province in the north east of Spain, includes a reference population of residents aged 25 to 74 years [22], [23]. There is one reference hospital with a coronary care unit, to which six community hospitals refer their AMI patients after emergency treatment.

Case-finding procedures were prospective for registering AMI patients admitted to the coronary care unit and retrospective in the community hospitals, accomplished by screening discharge records of all AMI patients they admitted. All discharges with International Classification of Diseases (ICD) codes 410–414 (for the 9^th^ revision) or I20–I25 (for the 10^th^ revision) were reviewed. In order to include out-of-hospital AMI events, all death certificates containing suggestive ICD codes (ICD-9: 410–414, 798; or ICD-10: I20–I25, I513, R960, R961, R98, R99) were investigated. To avoid case duplication the information from all sources was linked and verified by the researchers.

### Event classification

Each event was classified according to two different algorithms, applying the AHA/ESC [24] and the WHO-MONICA study definitions [16]. Both definitions are based on ECG findings, patient symptoms, and cardiac biomarkers. Data on symptoms, electrocardiographic variables and biomarkers were obtained from clinical records. Creatine-phosphokinase and troponins were used as biomarkers of myocardial necrosis in the WHO-MONICA and the AHA/ESC definitions, respectively. WHO/MONICA selected events were: fatal and non-fatal definite AMI, fatal possible AMI, and fatal cases with insufficient information [16]. Equivalent events were selected using the AHA/ESC algorithm: fatal and non-fatal definite AMI, fatal probable AMI, fatal possible coronary event, definite ischemic heart death, possible ischemic heart death, and fatal cases with insufficient information [24]. Two consecutive events in the same participant were considered as different events if the time between them exceeded 28 days.

### Study period

We included all registered AMI events from January 1, 2002 through December 31, 2008 from people aged 35–74 years residing in the study area. Two study periods were established: a 4-year pre-ban period (2002–2005) and a 3-year post-ban period (2006–2008). AMI events occurring in 2001 were excluded to diminish the impact of the AHA/ESC AMI redefinition and its gradual implementation on AMI incidence,

### Study variables

For this analysis the following variables were selected: age, sex, smoking, arterial hypertension, diabetes, hypercholesterolemia, and event-related variables such as symptoms, electrocardiographic variables, and biomarkers of myocardial necrosis. Data on hypercholesterolemia, hypertension, and smoking status were obtained by trained personnel using WHO-MONICA questionnaires. Current smokers were defined as persons who smoked more than 1 cigarette/day or reported quitting within the previous 12 months, while persons who had never smoked or smoked less than 1 cigarette/day were considered as non-smokers. Former smokers were defined as persons who had quit smoking more than 12 months before the event. Data on diabetes were obtained using WHO-MONICA questionnaires in addition to glucose values and treatment information obtained from clinical records. Validity of the WHO-MONICA questionnaires compared with clinical records has already been described [25], [26].

### Statistical analysis

We computed crude annual rates (×100,000 inhabitants) of population AMI incidence, AMI hospitalization, and population AMI mortality by age group (35–64 and 65–74 years) and sex. We also calculated AMI 28-day case-fatality. Separate calculations were done for events obtained with the AHA/ESC and the WHO-MONICA definitions. The rate denominator was taken from annual population distributions by sex and 5-year age groups published by the Statistical Institute of Catalonia [27].

Prior to conduct multivariate regression analyses, we analyzed data over-dispersion undertaking a likelihood ratio test where the null hypothesis was that the restriction implicit in the Poisson regression model was true (mean = variance). As this test was extremely significant (p-value = 2.2*10^−6^), we rejected the null hypothesis and assessed the effect of the partial smoke-free legislation on AMI incidence, mortality, and case-fatality by negative binomial regression analysis. The outcome variable was AMI events per quarter (for incidence models) or AMI fatal cases per quarter (for mortality and case-fatality models). The following predictor variables were included: age (categorized as 35–64 or 65–74 years), sex, and ban status (categorized as pre- or post-enactment). At-risk population by year, age group, and sex was included in the models as the offset variable in order to model the rate of events. In case-fatality models, at-risk events by year, age group, and sex were used as the offset variable. In addition, the models were adjusted for the existing trend and for seasonality. To adjust for the existing trend, we first assessed non-linearity of the existing time trend graphically and using a p-spline variable in an additive negative binomial regression model. As the graphs did not show a non-linear behavior and the spline variable was not significant (p>0.05), we used a linear variable to account for the existing trend. In order to adjust for seasonality, we used sine and cosine harmonic terms as previously described [7], [28]. The two harmonic terms that displayed the best adjustment based on the Akaike Information Criterion were selected: sine(ω*quarter) and cosine(ω*quarter), where ω = 2π/4. Relative risks and 95% confidence interval (CI) were obtained for each variable in the model.

We performed stratified analysis by age group, sex, and smoking status. For the latter, an approximate at-risk population served as the offset variable. This at-risk population was calculated using the percentage of smokers, non-smokers, and former smokers from two cross-sectional analyses in the same area in 2000 and 2005 [29]. Former and non-smokers were grouped in the same category as passive smokers.

To account for the missing values present in the smoking status variable we conducted a multiple imputation sensitivity analysis. Briefly, missing values were imputed using the following variables: age, sex, smoking status, hypertension, diabetes, hypercholesterolemia, previous AMI, vital status at 28 days, year and quarter. We obtained 20 multiple imputed databases for each AMI definition and we run in each one the described negative binomial regression model for smokers and passive smokers. Then, we calculated the global RR estimates and 95% confidence interval for each smoking group and definition.

Data analyses were performed using version 2.14.0 of the R statistical program (R Development Core Team (2011). R: A language and environment for statistical computing. R Foundation for Statistical Computing, Vienna, Austria. ISBN 3-900051-07-0, URL http://www.R-project.org/).

## Results

According to the AHA/ESC definition, a total of 3,703 AMI cases occurred in residents of the studied area between 2002 and 2008. Of those, 3,012 (81.3%) were admitted to hospital, and 2,142 of all AMI events (57.8%) occurred before the 2006 Spanish partial smoke-free legislation enactment. The characteristics of the overall population and of hospital-based cases are shown in Table 1, stratified by sex. Women were older and less likely than men to be smokers but with a higher proportion of diabetes and hypertension. These differences were observed at both the population and hospital levels. Hospital case-fatality was higher in women than in men. Demographic characteristics were similar when the WHO-MONICA AMI definition was used (Table S1).

**Table 1:** Characteristics of patients with AMI in Girona Province (Spain), in 2002–2008, according to the AHA/ESC AMI definition.

	Population-based registry cases				Hospital-based registry cases			
	Men	Women	Total	P value	Men	Women	Total	P value
**Events, n (%)**								
All cases	2895 (78.18)	808 (21.82)	3703 (100)		2341 (77.72)	671 (22.28)	3012 (100)	
35–64 years	1711 (59.10)	297 (36.80)	2008 (54.20)	<0.01	1450 (61.90)	262 (39.00)	1712 (56.80)	<0.01
65–74 years	1184 (40.90)	511 (63.20)	1695 (45.80)	<0.01	891 (38.10)	409 (61.00)	1300 (43.20)	<0.01
**Cardiovascular risk factors**								
Age (years, mean (SD))	60.87 (9.90)	64.67 (8.89)	61.25 (9.86)	<0.01	59.69 (9.97)	64.38 (9.01)	60.73 (9.96)	<0.01
Smoking, n (%)								
Current smoker	1211 (47.47)	131 (17.96)	1342 (40.91)	<0.01	1121 (49.17)	127 (19.48)	1248 (42.56)	<0.01
Former smoker	870 (34.20)	31 (4.25)	901 (27.46)	<0.01	763 (33.46)	29 (4.45)	792 (27.01)	<0.01
Never smoker	470 (18.42)	567 (77.77)	1037 (31.61)	<0.01	396 (17.37)	496 (76.07)	892 (30.42)	<0.01
Arterial hypertension, n (%)	1491 (58.90)	535 (72.80)	2026 (62.00)	<0.01	1339 (59.40)	480 (73.80)	1819 (62.60)	<0.01
Diabetes, n (%)	806 (32.50)	307 (42.10)	1113 (34.70)	<0.01	688 (31.20)	274 (42.20)	962 (33.70)	<0.01
Hypercholesterolemia, n (%)	1363 (55.90)	380 (53.70)	1743 (55.40)	0.31	1245 (57.60)	355 (56.60)	1600 (57.40)	0.68
Previous AMI, n (%)	558 (19.30)	140 (17.30)	698 (18.80)	0.18	430 (18.40)	121 (18.00)	551 (18.30)	0.93
**AMI Case-fatality, n (%)**	691 (23.86)	200 (24.75)	891 (24.06)	0.64	140 (6.00)	64 (9.50)	204 (6.80)	<0.01

*AMI* acute myocardial infarction, *AHA* American Heart Association, *ESC* European Society of Cardiology, *SD* Standard deviation.

**Figure 1:**
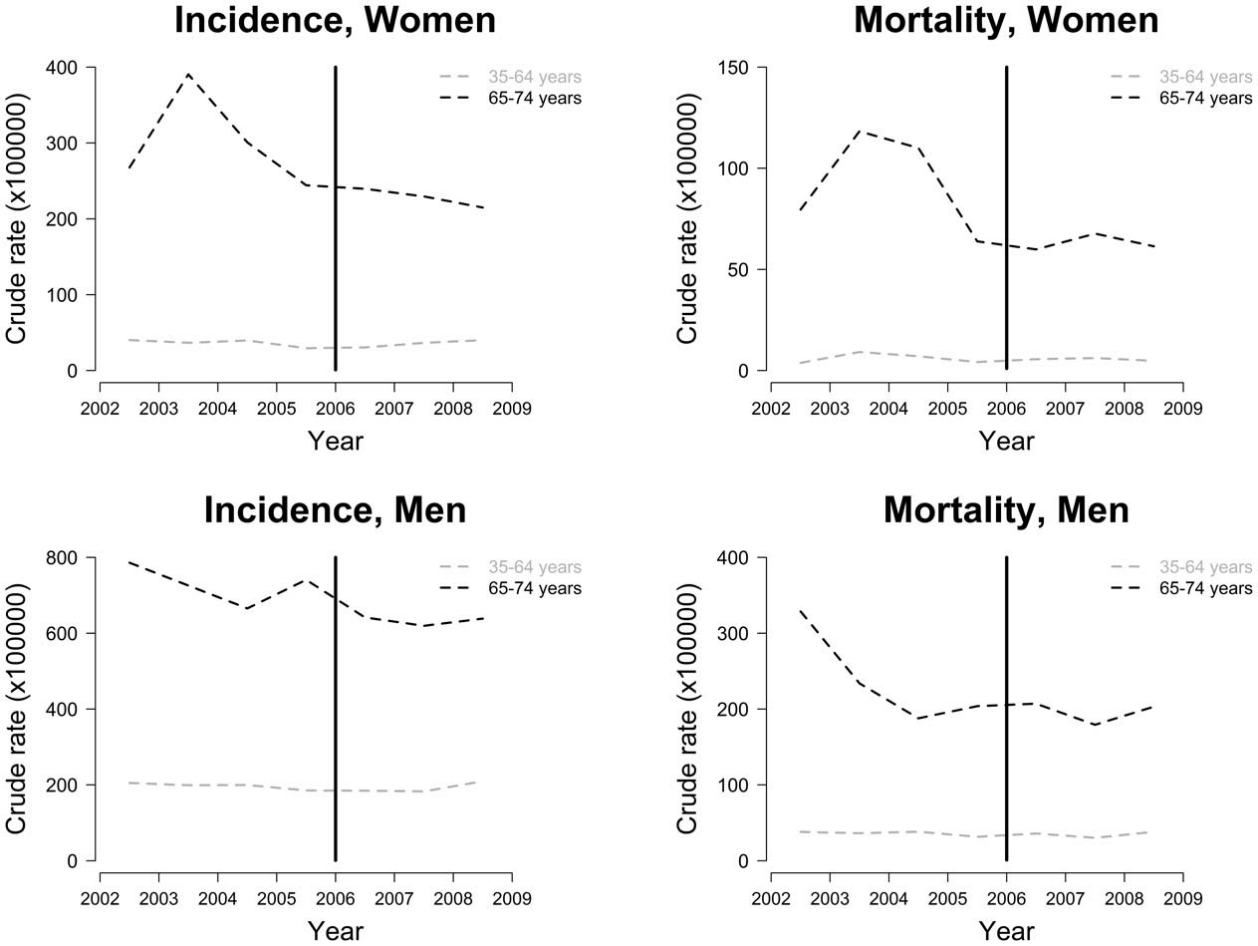
Annual AMI incidence and mortality crude rates by sex and age group. Time trend of the 2002–2008 AMI incidence and mortality crude rates according to the AHA/ESC AMI definition. Annual rates are plotted in the middle of each year. A vertical bar in 2006 indicates the start of the partial smoke-free legislation. Upper left panel: AMI incidence in women, upper right panel: AMI mortality in women, lower left panel: AMI incidence in men, lower right panel: AMI mortality in men. Abbreviations: *AMI* acute myocardial infarction, *AHA* American Heart Association, *ESC* European Society of Cardiology.

Table 2 shows the following annual crude rates using AHA/ESC-defined events: population AMI incidence, AMI hospitalization, and AMI mortality (×100,000) and 28-day AMI case-fatality, stratified by sex and age group from 2002 to 2008. Similar rates were found using the WHO-MONICA definition (Table S2). Time trends of crude incidence and mortality rates (according to the AHA/ESC AMI definition) for each sex and age group from 2002 to 2008 are presented in Figure 1.

**Table 2:** Annual AMI incidence, hospitalization and mortality rates and 28-day case-fatality by age group or sex, in 2002–2008, according to the AHA/ESC AMI definition.

Crude Cumulative AMI incidence rate (×100.000/year)							
	2002	2003	2004	2005	2006	2007	2008
Total	201.37	202.35	185.89	173.35	163.97	161.16	175.15
Women	87.84	108.76	90.34	68.91	67.58	69.44	70.39
Men	312.34	293.46	278.85	274.24	255.89	248.49	275.18
35–64 years	124.88	120.21	122.13	109.92	110.52	112.39	127.84
65–74 years	513.56	549.63	473.48	479.83	432.42	416.70	418.61

Crude AMI hospitalization rate (×100.000/year)							
	2002	2003	2004	2005	2006	2007	2008
Total	158.69	162.02	155.72	144.90	132.91	130.12	142.71
Women	74.96	86.85	74.68	58.57	58.96	54.00	59.76
Men	240.54	235.19	234.56	228.31	203.44	202.58	221.91
35–64 years	107.64	101.95	104.98	95.62	91.90	94.84	109.24
65–74 years	367.10	415.98	384.58	383.07	338.9	314.96	314.95

Crude AMI Mortality rate (×100.000/year)							
	2002	2003	2004	2005	2006	2007	2008
Total	56.14	51.85	45.60	37.58	39.46	35.12	39.57
Women	19.69	31.39	27.03	15.16	15.24	16.72	14.61
Men	91.78	71.77	63.66	59.24	62.55	52.64	63.41
35–64 years	21.44	23.15	23.14	18.39	21.33	18.67	22.08
65–74 years	197.82	173.17	146.88	130.32	130.51	121.29	129.57

AMI 28-day case-fatality (%)							
	2002	2003	2004	2005	2006	2007	2008
Total	27.88	25.62	24.53	21.68	24.06	21.79	22.59
Women	22.41	28.86	29.92	22.00	22.55	24.07	20.75
Men	29.38	24.46	22.83	21.6	24.44	21.18	23.04
35–64 years	17.16	19.26	18.95	16.73	19.3	16.61	17.27
65–74 years	38.52	31.51	31.02	27.16	30.18	29.11	30.95

*AMI* acute myocardial infarction, *AHA* American Heart Association, *ESC* European Society of Cardiology.

In the negative binomial regression analysis we observed a decrease in population AMI incidence in the post-ban period (Relative risk [RR]: 0.89; 95% CI: 0.81–0.97) using the AHA/ESC definition. This decrease was particularly significant among women (RR: 0.82), people aged 65–74 years (RR: 0.82) and passive smokers (RR: 0.85) (Table 3, upper left panel). When selecting hospital events only, the same general trend in AMI incidence emerged (RR: 0.89; 95% CI: 0.81–0.98), particularly significant for women (RR: 0.82) and people aged 65–74 years (RR: 0.83) (Table 3, upper right panel). Similar although slightly higher effects were observed when the WHO-MONICA definition was used (Table 3, lower panels). Estimates for smokers and passive smokers were also obtained by multiple imputation analysis to minimize the impact of missing data and similar results were observed (Table 3).

**Table 3:** RR and 95% CI for AMI incidence comparing the period after to the period before the 2006 partial smoke-free legislation enactment, according to both AHA/ESC and WHO-MONICA AMI definitions.

	Population-based registry events		Hospital-based registry events	
	Number of events	RR (95% CI)	Number of events	RR (95% CI)
**AHA/ESC**				
All	3703	0.89 (0.81–0.97)*	3011	0.89 (0.81–0.98)*
Women	808	0.82 (0.70–0.96)*	670	0.82 (0.70–0.96)*
Men	2895	0.93 (0.86–0.99)*	2341	0.93 (0.85–1.01)
<65years	2008	0.98 (0.89–1.07)	1712	0.96 (0.87–1.06)
≥65years	1695	0.82 (0.74–0.91)*	1299	0.83 (0.74–0.93)*
Smokers	1342	0.94 (0.83–1.06)	----	----
Passive smokers	1938	0.85 (0.76–0.95)*	----	----
MI Analysis				
SmokersPassive smokers		0.93 (0.82–1.05)0.88 (0.80–0.97)*		
**WHO-MONICA**				
All	3159	0.85 (0.77–0.94)*	2435	0.84 (0.77–0.93)*
Women	633	0.76 (0.64–0.90)*	484	0.75 (0.62–0.91)*
Men	2526	0.89 (0.82–0.97)*	1951	0.88 (0.80–0.97)*
<65years	1741	0.94 (0.85–1.04)	1436	0.92 (0.83–1.02)
≥65years	1418	0.78 (0.70–0.87)*	999	0.77 (0.67–0.88)*
Smokers	1173	0.91 (0.80–1.04)	----	----
Passive smokers	1579	0.78 (0.69–0.89)*	----	----
MI Analysis				
Smokers		0.91 (0.80–1.04)		
Passive smokers		0.82 (0.72–0.92)*		

Results from negative binomial regression analysis or multiple imputation (MI) analysis,

* p-value<0.05.

*RR* relative risk, *CI* confidence interval, *AMI* acute myocardial infarction, *AHA* American Heart Association, *ESC* European Society of Cardiology, *WHO* World Health Organization, *MONICA* Monitoring Trends and determinants in Cardiovascular diseases.

Using the AHA/ESC AMI definition, we found a general decrease in AMI population mortality in the post-ban period (RR: 0.82; 95% CI: 0.71–0.94), particularly significant for women (RR: 0.72) and people aged 65–74 years (RR: 0.74) (Table 4). Due to the inclusion of the same cases when using the AHA/ESC or the WHO-MONICA AMI definitions, the same AMI population mortality results were obtained with both definitions (Table 4, Table S3). In the case-fatality analysis, using all population events and the AHA/ESC AMI definition, we found a non-significant decrease in the post-ban period (Table 4). The association between the 2006 Spanish partial smoke-free legislation enactment and hospital-based mortality and 28-day case-fatality could not be analyzed due to an insufficient number of events to run the models. Due to the same reason, stratified analysis by smoking status could not be conducted in the hospital-based sample.

**Table 4:** RR and 95% CI for AMI mortality and 28-day case-fatality comparing the period after to the period before the 2006 partial smoke-free legislation enactment, according to the AHA/ESC AMI definition.

		Mortality	28-day case-fatality
	Number of events	RR (95% CI)	RR (95% CI)
All	891	0.82 (0.71–0.94)*	0.93 (0.81–1.06)
Women	200	0.72 (0.52–0.97)*	0.90 (0.67–1.21)
Men	691	0.85 (0.72–0.99)*	0.93 (0.80–1.09)
<65years	359	0.94 (0.76–1.17)	0.97 (0.78–1.20)
≥65years	532	0.74 (0.62–0.89)*	0.90 (0.75–1.08)

Results from negative binomial regression analysis.

* p-value<0.05.

*RR* relative risk, *CI* confidence interval, *AMI* acute myocardial infarction, *AHA* American Heart Association, *ESC* European Society of Cardiology.

## Discussion

The results of this population-based study show a decline in crude population AMI incidence and hospitalization in Girona Province after the 2006 Spanish partial smoke-free legislation enactment. This decrease was more pronounced among women, people aged 65–74 years and passive smokers. We also found a significant decrease in AMI mortality in women and people aged 65–74 years. However, no association was found between the partial smoke-free legislation enactment and AMI case-fatality.

The association between the implementation of a total smoke-free legislation and a reduction in hospitalization and population AMI incidence has already been described. Total smoke-free legislations have been estimated to decrease AMI hospitalization by 8–19% [30]–[33]. A lower decrease in hospital admissions is expected after the implementation of a partial smoke-free legislation, as this type of law will still allow smoking in certain places [33]. Our results show 11% decrease in AMI incidence and hospitalizations, similar to a recent report that showed 8.6% decrease in hospital admissions for AMI after a partial smoke-free legislation in Germany [34]. One possible explanation our estimate is not lower than the one reported from meta-analyses from total smoke-free legislations could be the duration of the post-ban period. We have included a 3-year post-ban period, one of the largest to date, and it has already been shown, that the larger the post-ban period, the larger the reduction in AMI incidence [30], [32], [34].

Most of the previous studies that analyzed the effect of the implementation of smoke-free legislation, used hospital-based AMI registries. Only two Italian studies used population-based registries [6], [7], with contradictory results: one reported a reduction in acute coronary events in Rome [6] and the other showed no association in the Tuscany population [7]. Gasparrini et al. [7] pointed out that the inconsistency between studies could be partially related to differences in the definition of the statistical model. Estimation of the effect of the bans is sensitive to the definition of the statistical model used to analyze the data, specifically the inclusion of the AMI incidence rate trend and the assumption of linearity of this trend. To prevent this potential bias, we tested the linearity of AMI incidence during the analyzed period, included an adjustment for the linear trend as appropriate, and also adjusted for seasonality.

Previous studies have yielded contradictory results regarding the effect of smoke-free legislations on AMI incidence and hospitalization rates by sex. Some described a higher decrease in men [6], [12], while others found a higher decrease in women [4], [9] or similar results in men and women [5], [8], [10]. Our study supports a higher decrease in AMI incidence and hospitalization rates in women than in men. It should be noted that the population incidence of AMI in women of Girona is among the lowest rates in the world [35]. The greater benefit in this population could be related to women's higher sensitivity to tobacco smoke compared to men. It has also been shown that smoking has a larger detrimental impact on myocardial infarction in women than in men [36], [37]. A plausible biological explanation for women's higher sensitivity to tobacco smoke is the alteration of lipid metabolism through the anti-estrogenic effect of smoking [38]–[41]. Estrogens have a beneficial effect on LDL- and HDL-cholesterol metabolism providing a protective effect against myocardial infarction in women [40], [41]. However, women who smoke are relatively estrogen deficient and have decreased levels of HDL-cholesterol [37], [38]. Our age group results, showing a larger effect in people aged >64 years, do not concur with previous studies showing a stronger AMI incidence/hospitalization decrease in people aged <60 or <70 years [4]–[6], [10]. We think that several factors are contributing to this difference. First, the 2006 partial smoke-free legislation banned smoking in all indoor public places and workplaces but allowed exceptions in hospitality venues. People aged 35–64 years will probably spend much more time at the places where smoking was still allowed (restaurants, bars, discos, etc) than people aged 65–74 years. Second, smoking sums up to the other risk factors, which are more prevalent in people aged 65–74 years. People aged 65–74 years also contribute more to the total number of AMI events than the 35–64 years group. Thus, a reduction in SHS exposure in the older age group would have a more profound effect, as this age group is at higher risk, and a reduction in one of the more significant risk factors would avoid a large number of cases.

In accordance with previous studies [8], [10], [11], we observed a decrease in AMI incidence/hospitalization rates among passive smokers, while no effect was observed in the smokers subgroup. Spanish partial smoke-free legislation led to a reduction in SHS exposure [42], [43], with a median decrease in nicotine concentration ranging from 60.0% in public places to 97.4% in private spaces, 96.7% in bars and restaurants that became smoke-free, and 88.9% in the no-smoking zones of venues with separate spaces for smokers [42]. Moreover, significant reductions on SHS were seen at individual [44] and population level [45]. However, no changes were seen neither in salivary cotinine concentration nor in self-reported exposure to SHS in workers at hospitality venues where smoking was not totally banned [44]. Reduction in SHS exposure has also been reported in other populations after a smoke-free legislation [46]. All these data suggest that reduced SHS exposure was the fundamental underlying factor explaining the health effects of the smoke-free legislation.

Our results also showed an association between the Spanish partial smoke-free legislation and a reduction in AMI mortality, particularly in the population aged 65–74 years. Four studies have analyzed AMI mortality during a smoke-free legislation enactment, 3 in the USA [17]–[19] and 1 in Spain [20], showing a reduction in AMI mortality only when a post-ban period longer than 2 years was considered [17], [20]. Our study analyzes this trend over the longest post-ban period reported to date and contributes to the evidence of an association between reduced AMI mortality and the enactment of a smoke-free legislation. We also found a non-significant decrease in AMI case-fatality after the partial smoke-free legislation enactment, indicating that the decrease in AMI mortality is mainly due to the decrease in incidence.

The effect sizes of the reported associations are very important from a public health perspective. The post-ban 18% AMI mortality decrease observed in the population aged 35–74 years is comparable with the reported effect of blood pressure control on coronary heart disease mortality in the 1988–2005 period in Spain [47].

To avoid the effect of including different definitions of AMI over time, we adhered to the new AHA/ESC definition algorithms [24] and excluded 2001 events to avoid the effect of the gradual implementation of the new definition. In parallel we used the classical WHO-MONICA AMI definition [16], which ignores the troponin values. We observed that although the results are concordant, the effect of the 2006 Spanish partial smoke-free legislation was slightly higher when using the latter definition. This difference could explain some of the inconsistencies observed in previous reports.

Our study has several strengths. First, we used population-based data drawn from an AMI registry operating since 1990, which included all hospitalized cases and out-of-hospital deaths. Second, the availability of individual patient-level information such as smoking status permitted us to analyze subgroups of the population. Third, the present study is the first to present a parallel analysis using both AHA/ESC and WHO-MONICA AMI definitions. This approach increases the robustness of our results, bypassing the impact of using the AHA/ESC AMI redefinition alone, which could have introduced a bias. Finally, our data were collected with identical methods over a long pre- and post-ban period, increasing the stability of the estimations.

Our study also has a number of limitations that should be considered. First, due to the ecological nature of the study, no causal relationship can be inferred between the implementation of the 2006 Spanish partial smoke-free legislation and the reduction in AMI incidence and mortality. Second, unmeasured potential confounding factors, such as patient comorbidities and AMI case severity, other activities affecting smoking behavior, changes in AMI prevention and treatment strategies, and changes in air quality may have operated together with the partial smoke-free legislation introduced in 2006. Third, the study does not include direct observations on SHS exposure and consequently, despite the reported statistical associations, it is not possible to confirm a reduction in individual SHS exposure during the months following the ban enactment.

## Conclusions

The Spanish 2006 partial smoke-free legislation was associated with a reduction in AMI population incidence, mortality, and hospitalization rates but was not associated with AMI case-fatality in Girona Province. These results support the effectiveness of smoking regulations as public health interventions that prevent CHD, particularly in passive smokers. Further studies are needed to confirm the impact and effectiveness of the 2011 Spanish total smoke-free legislation.

## Supporting Information

Table S1 Characteristics of patients with AMI in Girona Province (Spain), according to the WHO-MONICA AMI definition.
(DOC)

Table S2 Annual AMI incidence, hospitalization and mortality crude rates and 28-day case-fatality by age group or sex, according to the WHO-MONICA AMI definition.
(DOC)

Table S3 RR and 95% CI for AMI mortality and 28-day case-fatality comparing the period after to the period before the 2006 smoking ban enactment, according to the WHO-MONICA AMI definition.
(DOC)
