# Supplementary material for: Impact of a Partial Smoke-Free Legislation on Myocardial Infarction Incidence, Mortality and Case-Fatality in a Population-Based Registry: The REGICOR Study
Source: PLoS One. 2013 Jan 23;8(1):e53722. doi: 10.1371/journal.pone.0053722 (PMC3553094; doi:10.1371/journal.pone.0053722)
Supplement: Table S1 — Characteristics of patients with AMI in Girona Province (Spain), according to the WHO-MONICA AMI definition. (DOC) [file pone.0053722.s001.doc]

**Supplementary table 1.** Characteristics of patients with AMI in Girona Province (Spain), according to the WHO-MONICA AMI definition.

|  | **Population-based registry cases** | | | | **Hospital-based registry cases** | | | |
| --- | --- | --- | --- | --- | --- | --- | --- | --- |
|  | **Men** | **Women** | **Total** | **P value** | **Men** | **Women** | **Total** | **P value** |
| **Events, n (%)** |  |  |  |  |  |  |  |  |
| All cases | 2526 (79.96) | 633 (20.04) | 3159 (100) |  | 1998 (79.82) | 505 (20.18) | 2503 (100) |  |
| 35-64 years | 1514 (59.94) | 227 (35.86) | 1741 (55.11) | <0.01 | 1263 (63.21) | 193 (38.22) | 1456 (58.17) | <0.01 |
| 65-74 years | 1012 (40.06) | 406 (64.14) | 1418 (44.89) | <0.01 | 735 (36.79) | 312 (61.78) | 1047 (41.83) | <0.01 |
| **Cardiovascular risk factors** |  |  |  |  |  |  |  |  |
| Age (years, mean (SD)) | 60.02 (9.95) | 64.7 (9.14) | 60.96 (9.97) | <0.01 | 59.19 (9.03) | 64.02 (9.40) | 60.15 (10.09) |  |
| Smoking, n (%) |  |  |  |  |  |  |  |  |
| Current smoker | 1063 (46.06) | 110 (18.68) | 1173 (40.49) | <0.01 | 967 (50.87) | 106 (22.41) | 1073 (45.20) | <0.01 |
| Former smoker | 841 (36.44) | 46 (7.81) | 887 (30.62) | <0.01 | 607 (31.93) | 16 (3.38) | 623 (26.24) | <0.01 |
| Never smoker | 404 (17.50) | 433 (73.51) | 837 (28.89) | <0.01 | 327 (17.20) | 351 (74.21) | 678 (28.56) | <0.01 |
| Arterial hypertension, n (%) | 1252 (57.54) | 389 (68.85) | 1641 (59.87) | <0.01 | 1086 (57.80) | 324 (69.23) | 1410 (60.08) | <0.01 |
| Diabetes, n (%) | 666 (31.37) | 230 (40.93) | 896 (33.37) | <0.01 | 543 (29.69) | 189 (40.30) | 732 (31.85) | <0.01 |
| Hypercholesterolemia, n (%) | 1162 (55.54) | 284 (51.92) | 1446 (54.79) | 0.14 | 1029 (57.23) | 253 (55.73) | 1282 (56.93) | 0.60 |
| Previous AMI, n (%) | 457 (18.09) | 94 (14.85) | 551 (17.44) | 0.05 | 321 (16.45) | 70 (14.46) | 391 (16.06) | 0.31 |
| **AMI Case-fatality, n (%)** | 692 (27.40) | 200 (31.60) | 892 (28.24) | 0.04 | 119 (6.10) | 52 (10.74) | 171 (7.02) | <0.01 |

Registered cases from 2002 to 2008.

*AMI* acute myocardial infarction, *WHO* World Health Organization, *MONICA* Monitoring Trends and determinants in Cardiovascular diseases, *SD* Standard deviation
