# Supplementary material for: Impact of a Partial Smoke-Free Legislation on Myocardial Infarction Incidence, Mortality and Case-Fatality in a Population-Based Registry: The REGICOR Study
Source: PLoS One. 2013 Jan 23;8(1):e53722. doi: 10.1371/journal.pone.0053722 (PMC3553094; doi:10.1371/journal.pone.0053722)
Supplement: Table S2 — Annual AMI incidence, hospitalization and mortality crude rates and 28-day case-fatality by age group or sex, according to the WHO-MONICA AMI definition. (DOC) [file pone.0053722.s002.doc]

**Supplementary table 2.** Annual AMI incidence, hospitalization and mortality crude rates and 28-day case-fatality by age group or sex, according to the WHO-MONICA AMI definition.

| **Crude Cumulative AMI incidence rate (x 100.000/year)** | | | | | | | |
| --- | --- | --- | --- | --- | --- | --- | --- |
|  | **2002** | **2003** | **2004** | **2005** | **2006** | **2007** | **2008** |
| Total | 141.11 | 139.34 | 129.77 | 116.81 | 105.11 | 105.35 | 117.74 |
| Women | 73.45 | 91.97 | 69.71 | 50.99 | 50.35 | 52.08 | 53.79 |
| Men | 286.44 | 260.77 | 243.56 | 233.64 | 215.45 | 212.99 | 240.94 |
| 35-64 years | 113.23 | 107.30 | 106.70 | 91.94 | 93.07 | 96.34 | 110.41 |
| 65-74 years | 458.40 | 474.34 | 388.45 | 394.92 | 344.77 | 334.53 | 350.84 |
| **Crude AMI hospitalization rate (x 100.000/year)** | | | | | | | |
|  | **2002** | **2003** | **2004** | **2005** | **2006** | **2007** | **2008** |
| Total | 136.24 | 134.66 | 126.27 | 114.10 | 102.20 | 103.47 | 114.82 |
| Women | 60.58 | 67.15 | 51.21 | 39.28 | 41.08 | 36.65 | 42.50 |
| Men | 210.20 | 200.37 | 199.28 | 186.38 | 160.49 | 167.09 | 183.88 |
| 35-64 years | 95.06 | 87.71 | 89.56 | 77.64 | 73.68 | 79.16 | 90.65 |
| 65-74 years | 304.33 | 333.16 | 291.82 | 290.27 | 245.43 | 230.85 | 239.21 |
| **Crude AMI Mortality rate (x100.000/year)** | | | | | | | |
|  | **2002** | **2003** | **2004** | **2005** | **2006** | **2007** | **2008** |
| Total | 56.14 | 51.85 | 45.6 | 37.58 | 39.46 | 35.12 | 39.90 |
| Women | 19.69 | 31.39 | 27.03 | 15.16 | 15.24 | 16.72 | 14.61 |
| Men | 91.78 | 71.77 | 63.66 | 59.24 | 62.55 | 52.64 | 64.04 |
| 35-64 years | 21.44 | 23.15 | 23.14 | 18.39 | 21.33 | 18.67 | 22.47 |
| 65-74 years | 197.82 | 173.17 | 146.88 | 130.32 | 130.51 | 121.29 | 129.57 |
| **AMI 28-day case-fatality (%)** | | | | | | | |
|  | **2002** | **2003** | **2004** | **2005** | **2006** | **2007** | **2008** |
| Total | 30.99 | 29.21 | 28.89 | 26.12 | 29.26 | 26.11 | 26.68 |
| Women | 26.80 | 34.13 | 38.78 | 29.73 | 30.26 | 32.10 | 27.16 |
| Men | 32.04 | 27.52 | 26.14 | 25.36 | 29.03 | 24.71 | 26.58 |
| 35-64 years | 18.93 | 21.58 | 21.69 | 20.00 | 22.92 | 19.38 | 20.35 |
| 65-74 years | 43.15 | 36.51 | 37.81 | 33.00 | 37.85 | 36.26 | 36.93 |

*AMI* Acute myocardial infarction, *WHO* World Health Organization, *MONICA* Monitoring Trends and determinants in cardiovascular diseases
