# Supplementary material for: Impact of a Partial Smoke-Free Legislation on Myocardial Infarction Incidence, Mortality and Case-Fatality in a Population-Based Registry: The REGICOR Study
Source: PLoS One. 2013 Jan 23;8(1):e53722. doi: 10.1371/journal.pone.0053722 (PMC3553094; doi:10.1371/journal.pone.0053722)
Supplement: Table S3 — RR and 95% CI for AMI mortality and 28-day case-fatality comparing the period after to the period before the 2006 smoking ban enactment, according to the WHO-MONICA AMI definition. (DOC) [file pone.0053722.s003.doc]

**Supplementary table 3.** RR and 95% CI for AMI mortality and 28-day case-fatality comparing the period after to the period before the 2006 smoking ban enactment, according to the WHO-MONICA AMI definition.

|  |  | **Mortality** | **28-day case-fatality** |
| --- | --- | --- | --- |
|  | **Number of events** | **RR (95% CI)** | **RR (95% CI)** |
| All | 892 | 0.82 (0.71-0.95)* | 0.98 (0.85-1.12) |
| Women | 200 | 0.72 (0.52-0.97)* | 0.98 (0.72-1.31) |
| Men | 692 | 0.85 (0.73-0.99)* | 0.98 (0.86-1.14) |
| < 65years | 360 | 0.95 (0.77-1.18) | 1.01 (0.81-1.25) |
| ≥ 65years | 532 | 0.74 (0.62-0.89)* | 0.96 (0.80-1.14) |

Results from negative binomial regression analysis

* p-value < 0.05

*RR* relative risk, *CI* confidence interval, *AMI* acute myocardial infarction, *WHO* World Health Organization, *MONICA* Monitoring Trends and determinants in Cardiovascular diseases
